# miR-638 promotes melanoma metastasis and protects melanoma cells from apoptosis and autophagy

**DOI:** 10.18632/oncotarget.3070

**Published:** 2014-12-26

**Authors:** Animesh Bhattacharya, Ulf Schmitz, Yvonne Raatz, Madeleine Schönherr, Tina Kottek, Marianne Schauer, Sandra Franz, Anja Saalbach, Ulf Anderegg, Olaf Wolkenhauer, Dirk Schadendorf, Jan C. Simon, Thomas Magin, Julio Vera, Manfred Kunz

**Affiliations:** ^1^ Department of Dermatology, Venereology and Allergology, University of Leipzig, Leipzig, Germany; ^2^ Department of Systems Biology and Bioinformatics, University of Rostock, Rostock, Germany; ^3^ Department of Dermatology, Venereology and Allergology, University Hospital Essen, Essen, Germany; ^4^ Institute of Biology and Translational Centre for Regenerative Medicine (TRM), University of Leipzig, Leipzig, Germany; ^5^ Laboratory of Systems Tumor Immunology, Department of Dermatology, Faculty of Medicine, University of Erlangen-Nuremberg, Erlangen, Germany

**Keywords:** Melanoma, metastasis, microRNA, p53 pathway, apoptosis

## Abstract

The present study identified miR-638 as one of the most significantly overexpressed miRNAs in metastatic lesions of melanomas compared with primary melanomas. miR-638 enhanced the tumorigenic properties of melanoma cells *in vitro* and lung colonization *in vivo*. mRNA expression profiling identified new candidate genes including *TP53INP2* as miR-638 targets, the majority of which are involved in p53 signalling. Overexpression of *TP53INP2* severely attenuated proliferative and invasive capacity of melanoma cells which was reversed by miR-638. Depletion of miR-638 stimulated expression of p53 and p53 downstream target genes and induced apoptosis and autophagy. miR-638 promoter analysis identified the miR-638 target transcription factor associated protein 2α (TFAP2A/AP-2α) as a direct negative regulator of miR-638, suggestive for a double-negative regulatory feedback loop. Taken together, miR-638 supports melanoma progression and suppresses p53-mediated apoptosis pathways, autophagy and expression of the transcriptional repressor TFAP2A/AP-2α.

## INTRODUCTION

Melanoma is a malignant tumor of high metastatic potential [1]. Activating mutations in *BRAF* and *NRAS* oncogenes have been identified in a majority of melanoma patients and appear to play an important role in its pathogenesis, with both being mutually exclusive [2]. However, a majority of patients with mutated *BRAF* and initial treatment response to specific BRAF inhibitors like vemurafenib develop recurrences due to a variety of different mechanisms, including secondary *NRAS* mutations, *BRAF* amplifications and enhanced PDGFR expression [3,4]. This emphasizes the need to further improve our understanding of the process of tumor metastasis and to identify new targetable oncogenes.

Experimental evidences accumulated over the past decade demonstrate the importance of microRNA (miRNA) dysregulation for tumor development and progression [5]. Regarding melanoma, expression profiling studies of melanoma samples have identified a number of oncogenic miRNAs (oncomirs), like miR-214, miR-182, and miR-30b/30d, the expression of which correlates with both disease progression and outcome [6,7,8]. Tumors may even become dependent on oncomirs and are unable to adapt to its depletion. This phenomenon was termed as oncomir addiction [9,10]. This suggests that oncomirs may be effective targets for tumor therapies.

In the present report, the role of miRNAs in regulation of the metastatic process in melanoma was investigated.

## RESULTS

### miR-638 is strongly upregulated during melanoma progression

**Figure 1:**
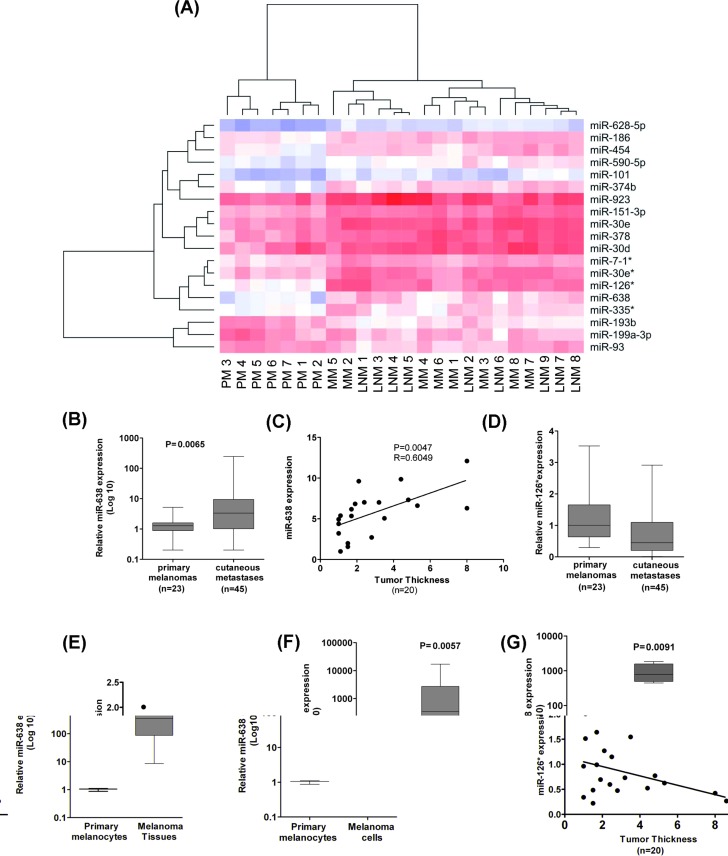
Expression of miR-638 directly correlates with melanoma progression (A) Heatmap of differentially expressed miRNAs in melanoma patient tissue samples comprising primary melanomas, lymph node metastases and distant cutaneous metastases (ou) (n=24), generated using expression data from TaqMan® low density miRNA arrays. Colors represent normalized *Ct* values (blue for high *Ct* values indicates low expression and red for low *Ct* values indicates high expression of miRNAs). Dendrograms are based on hierarchical clustering with Euclidean distance. (B) miR-638 expression in PM and MM and (C) PM of varying thickness (mm). (D) miR-126* expression in PM and MM and (E) PM of varying thickness (mm). Paired *t-*test and Pearson's coefficient (Pearson's r) were used to determine significance of correlation between miR-638 expression and tumor thickness. (F, G) Expression levels of miR-638 were analysed in primary melanocytes (n=3) in comparison with (F) PM and MM tissue samples (n=27). (G) miR-638 expression in 9 different melanoma cell lines (HT144, 1F6, Mewo, SK-Mel-147, SK-Mel-19, SK-Mel-28, SK-Mel-29, A375 and BRO). All miRNA expression analyses were performed using TaqMan® qRT-PCR technology. RNU48 expression was used as universal reference control for miRNAs.

The expression of 667 different miRNAs was analyzed in primary melanomas (PM), lymph node metastases (LNM) and distant cutaneous metastases (MM), respectively. There was little correlation between miRNA expression profiles of PMs, which may be due to the known genetic heterogeneity of primary tumors. In contrast, miRNA profiles of LNMs and MMs correlated well and were clearly separated from those of primary tumors, arguing for a closer relationship between metastatic lesions (Supplementary Fig. S1). Thus, miRNA patterns may characterize different stages of melanoma progression. In total, 18 miRNAs were shown to be differentially expressed between the primary melanoma samples and metastases samples (Fig. 1A, Supplementary Table S1). miR-126* (mir-126-5p) and miR-638 were the top two upregulated candidates in metastatic samples as compared with primary melanomas. Analysis in a separate set of PM and MM samples confirmed upregulation (11.8 fold) of miR-638 in MMs (Fig. 1B, Supplementary Table S2). miR-638 expression exhibited a positive correlation with primary tumor thickness, which is the major prognostic factor for melanoma (Fig. 1C). miR-126* expression did not correlate with the primary tumor thickness and showed variable expression levels across different MM and PM samples (Fig. 1D, E). Interestingly, primary melanocytes exhibited extremely low expression of miR-638 as compared with melanoma samples and melanoma cell lines (Fig. 1F, G).

Taken together, these findings suggest that miR-638 upregulation is associated melanoma initiation and progression.

### miR-638 overexpression enhances the tumorigenic and metastatic potential of melanoma cells

**Figure 2:**
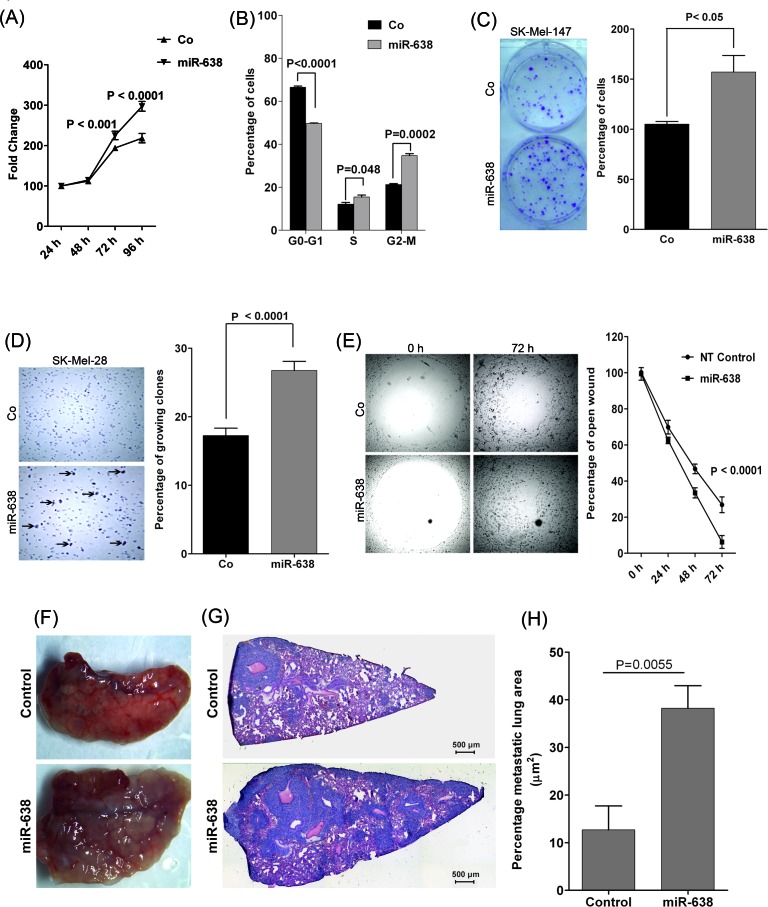
miR-638 promotes tumorigenic and metastatic properties of melanoma cells *in vitro* and *in vivo* (A) XTT cell proliferation assay using SK-Mel-28 cells transiently overexpressing a scrambled-control or miR-638 (mean ± S.E.M; n=3). (B) Cell cycle analysis for SK-Mel-28 cells transiently overexpressing a scrambled-control or miR-638. (mean ± S.E.M.; n=3) (C) Colony formation assay after low density seeding of SK-Mel-147 cells overexpressing a scrambled-control or miR-638 (mean ± S.E.M; n=3). (D) Soft agar assays using SK-Mel-28 cells transiently overexpressing a scrambled-control or miR-638 (mean ± S.E.M; n=3). Arrows indicate colonies of growing cells. (E) Migration assays were performed for SK-Mel-147 cells transiently overexpressing a scrambled-control or miR-638. Microscopic pictures were taken at indicated time points (mean ± S.E.M, n=3). NT; scrambled. (F-H) *In vivo* metastasis (lung colonization) experiments with stably miR-638 transduced or control transduced SK-Mel-147 cells injected into the lateral tail vein of NSG mice (n=5 per group). (F) Macroscopic pictures of mouse lungs 21 days after tail vein injection. (G) Hematoxylin&Eosin (H&E)-stained sections of lung metastases (right; magnification x4). (H) Percentage of lung area covered by metastatic tumor areas analysed in the H&E stained lung sections. A minimum of 3 lung sections per mouse were quantitated (mean ± S.E.M.). All biological assays were performed in triplicates and repeated twice upon individual transfections and assay measurement.

miR-638 is encoded on chromosome 19p13.2 in the intronic region of *DNM2*, which is reported to be an oncogene [Supplementary Fig. S2] [11,12]. To further address the role of miR-638 in melanoma, its influence was analysed on SK-Mel-28 and SK-Mel-147 melanoma cells which express moderate levels of miR-638 (Supplementary Fig. S3A). Overexpression of miR-638 significantly increased proliferation of otherwise slowly proliferating SK-Mel-28 melanoma cells (Fig. 2A). Moreover, miR-638 significantly enhanced the progression of melanoma cells through the cell cycle (G1-S-G2/M) (Fig. 2B). Furthermore, the clonogenic potential of melanoma cells significantly increased (1.5 fold) upon miR-638 overexpression (Fig. 2C, Supplementary Fig. S3B). In an attachment-independent soft agar assay, miR-638 overexpressing melanoma cells exhibited significantly higher (1.55 fold) capability to form multicellular clones (Fig. 2D). Additionally, in an *in vitro* migration assay, miR-638-transfected SK-Mel-147 and SK-Mel-28 melanoma cells were more efficient than control cells in closing an artificial wound created over a confluent monolayer of cells (Fig. 2E, Supplementary Fig. S3C). In an *in vitro* transwell invasion assay, higher percentage of miR-638 transfected SK-Mel-147 cells were able to migrate through the matrigel coated membrane as compared to the control cells (Supplementary Fig. S3D).

Next, we asked the question whether miR-638 host gene *DNM2* contributes to the pro-tumorigenic and metastatic effects of miR-638. *DNM2* knockdown did not exhibit any inhibitory effect on migration of melanoma cells (Supplementary Fig. S3E,). Moreover, endogenous depletion of miR-638 but not of *DNM2* significantly attenuated the proliferation of melanoma cells (Supplementary Fig. S3G).

These *in vitro* results prompted *in vivo* studies regarding the role of miR-638. SK-Mel-147 cells, stably over-expressing miR-638 (shMIMIC-miR-638) or a scrambled control were injected into the tail veins of 8-week-old NSG (NOD *scid* IL-2 receptor gamma chain knockout) mice. 21 days after injection, lungs surgically removed from mice injected with miR-638-overexpressing cells macroscopically displayed significantly more metastatic nodules than lungs of mice injected with control transduced cells (Fig. 2F). Lung sections of mice injected with miR-638-transduced cells in fact showed almost three times the size of metastatic tumor areas and significantly higher number of metastatic colonies as compared with control-injected mice (Fig. 2G, H, Supplementary Fig. S3H).

These results clearly demonstrate that miR-638 augments the proliferative and metastatic capacities of melanoma cells.

### miR-638 mediated repression of TP53INP2 facilitates tumor progression

**Figure 3:**
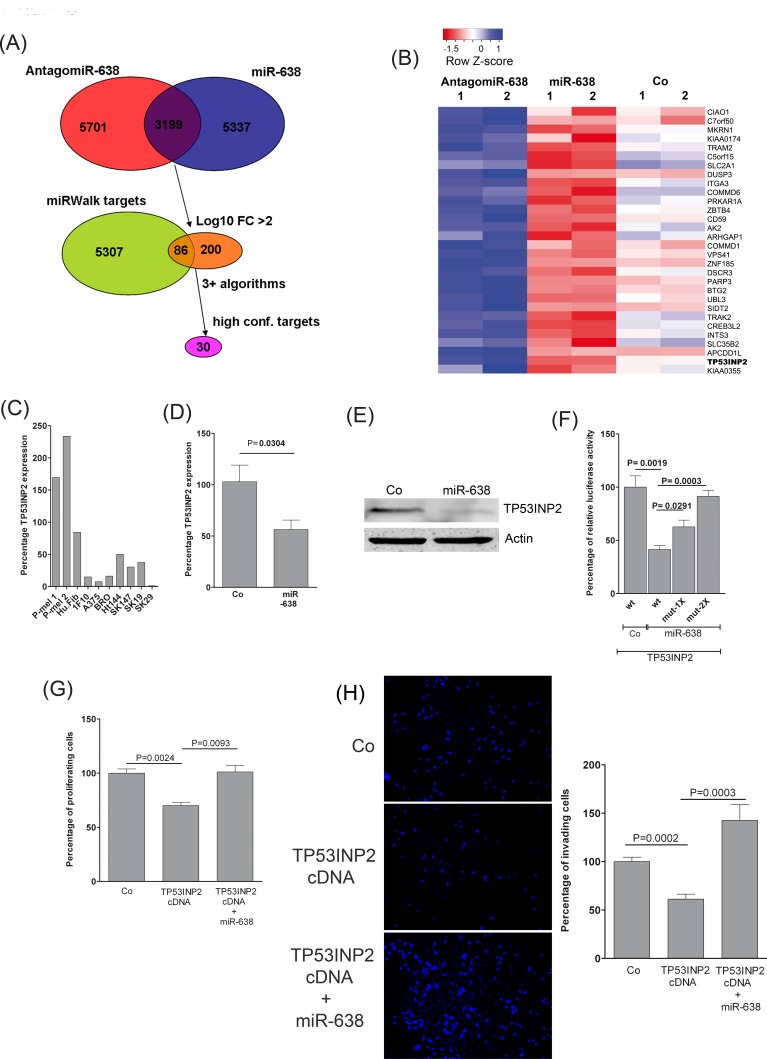
miR-638 promotes proliferation and invasion by directly targeting TP53INP2 (A) Melanoma cells were transiently transfected with miR-638, antagomir-638 or control-antagomiR and analyzed using whole-genome cDNA microarrays. The Venn diagram at the top displays the overlap between down-regulated and up-regulated genes upon miR-638 and antagomiR-638 overexpression, respectively (n = 3,199). The middle Venn diagram displays the intersection of miRWalk database predicted miR-638 targets and strongly differentially regulated mRNAs (total log_10_ fold change (FC) > 2; n = 86). Finally, a subset of targets predicted by at least three different algorithms as high-confidence targets of miR-638 regulation was identified and used in subsequent analyses (n = 30). (B) Heatmap of differentially regulated putative target genes for miR-638. Colors represent fold changes of relative gene expressions of miR-638 or antagomiR-638-transfected cells in comparison with mock transfected control cells (blue color indicates high expression and red color indicates low expression). (C) *TP53INP2* mRNA expression analysis for primary melanocytes (P-mel 1 and 2), human fibroblasts (Hu.Fib), and melanoma cell lines, respectively, using TaqMan® gene expression assays. Normalized *Ct* values were quantitated and compared with the mean *TP53INP2* expression values, respectively, across all cell lines. Results are expressed as percentage of relative expression. (D) SK-Mel-147 cells were transfected with a scrambled-control (Co) or miR-638. Fourty eight hours after transfection *TP53INP2* mRNA expression was analysed using TaqMan® gene expression assays. GAPDH was used as reference control (mean ± S.E.M). (E) SK-Mel-147 melanoma cells were transfected with scrambled-control (Co) or miR-638. After 48 h of transfection, protein expression for TP53INP2 was analysed using immunoblotting. β-actin was used as loading control. (F) Reporter assay in SK-Mel-147 cells co-transfected with a scrambled miRNA control or miR-638 and with either wild type or single (1X) or double (2X) mutated *TP53INP2* 3′-UTR cloned in a dual-luciferase constructs (mean ± S.E.M; n= 4). (G) XTT cell proliferation assay using SK-Mel-147 cells transiently overexpressing control plasmid or *TP53INP2* cDNA co-transfected with a control miRNA or miR-638 (mean ± S.E.M; n=3). The UV-absorptions were measured at 24 h and 48 h at 492 nm (H) Matrigel invasion assays were performed for SK-Mel-147 cells transiently overexpressing a control plasmid or *TP53INP2* cDNA co-transfected with a control miRNA or miR-638. Microscopic pictures were taken at 48 h (mean ± S.E.M, n=3). All biological assays were performed in triplicates and repeated twice individual transfections and assay measurements.

To identify miR-638 target genes which might account for its oncogenic and pro-metastatic properties in melanoma, mRNA expression profiling was performed using commercially available cDNA microarrays. For this purpose, SK-Mel-147 cells were transfected with miR-638 or antagomiR-638 oligomers and compared with the mock transfected control cells. From the list of differentially expressed genes, only those genes were considered as candidate miR-638 target genes that were at the same time significantly suppressed by miR-638 and de-repressed by antagomiR-638 (Fig. 3A). To identify the most probable direct targets for miR-638, downregulated genes were compared with predicted miR-638 targets using eight renowned miRNA-target prediction algorithms extracted from the miRWalk database [13]. Remarkably, among the top predicted miR-638 targets (predicted by at least 5 miRNA-databases), TP53-inducible nuclear protein 2 (TP53INP2) expression was the most strongly influenced both upon miR-638 overexpression and knockdown. Therefore, *TP53INP2* was selected for further analysis (Fig. 3B, Supplementary Fig. S4, Supplementary Table S3).

The expression of TP53INP2 was markedly reduced in most of the melanoma cell lines as compared to benign cells (Fig. 3C). Moreover, the mRNA and protein levels of TP53INP2 in melanoma cells were significantly suppressed upon transfection of miR-638 (Fig. 3D, E). This suggests that TP53INP2 is transcriptionally regulated by miR-638. Using 3′-UTR luciferase reporter gene assays, it was verified that *TP53INP2* is a direct target for miR-638. Furthermore, mutations in the miR-638 binding sites in the 3′-UTR of *TP53INP2* were able to reverse miR-638 mediated TP53INP2 repression (Fig. 3F). Overexpression of *TP53INP2* cDNA in melanoma cells resulted in significant attenuation of their proliferative and invasive potential which was rescued upon miR-638 co-transfection (Fig. 3G, H).

Overall, these results suggest that miR-638 induces its pro-tumorigenic and metastatic effects at least in part by repressing the identified targets like TP53INP2.

### Knockdown of miR-638 induces p53-mediated apoptosis and autophagy in melanoma cells

**Figure 4:**
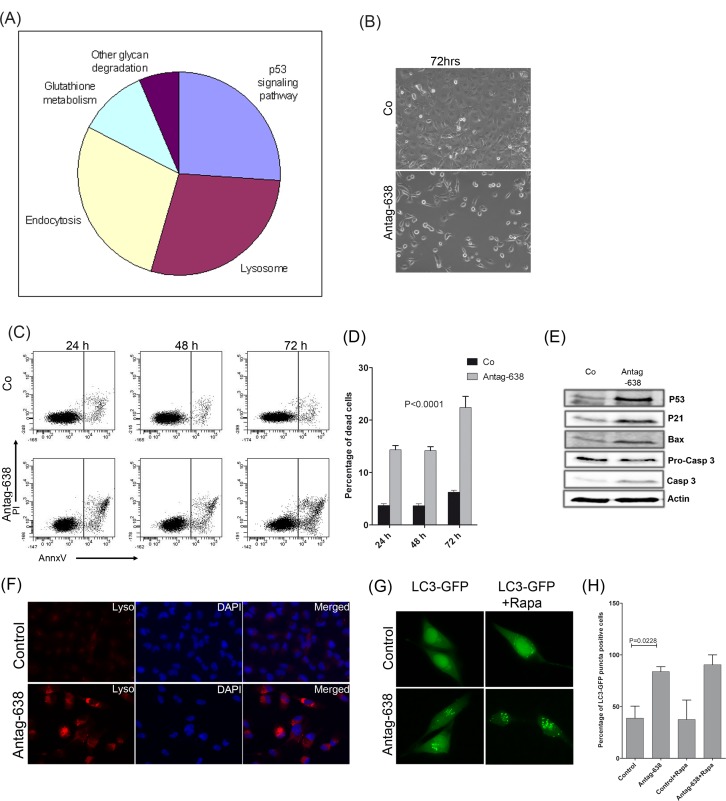
Knockdown of miR-638 induces p53-mediated apoptosis and autophagy (A) Genes de-repressed by antagomiR-638 treatment of melanoma cells were subjected to functional enrichment analysis, using DAVID bioinformatics tool. Different pathway candidates regulated by miR-638, including p53 signalling and lysosomal pathways, were identified. (B) Microscopic pictures of SK-Mel-147 cells overexpressing control-antagomiR (Co) or antagomiR-638 (Antag-638), showing confluency of melanoma cells after 72 h of transfection. (C, D) Human SK-Mel-147 melanoma cells were transfected with control-antagomiR (Co), or antagomiR-638 (Antag-638). At indicated time points, cells were stained for annexin-V/propidium iodide (PI), and apoptotic cells were analysed by flow cytometry. (C) Representative histograms indicating the proportion of annexin V/PI-stained and unstained SK-Mel-147 cells. (D) Graphs indicate percentage of dead cells (mean ± S.E.M). (E) Human SK-Mel-147 cells were transfected with control-antagomiR, antagomiR-638. Expression levels of the indicated apoptosis-related proteins were analysed by immunoblotting. (F) Fluorescent lysosomal staining (lysotracker red) of SK-Mel-147 cells transfected with control-antagomiR or antagomiR-638. Cell nuclei are stained with DAPI (x40). (G) SK-Mel-28 cells co-transfected with an EGFP-LC3B containing construct and a control-antagomiR or antagomiR-638. After 24 h of transfection cells were either left untreated or treated with 100 nM rapamycin for another 24 h, after which they were fixed and microscopic images were taken (x60). (H) The percentage of EGFP-LC3B puncta-positive cells was quantitated (mean ± S.E.M). All biological assays were performed in triplicates and repeated twice upon individual transfections.

To gain a better mechanistic insight into the pathways regulated by miR-638, the list of target genes de-repressed by antagomiR-638 (≥1.5 fold) was subjected to a functional enrichment analysis using DAVID (Supplementary Table S4). Interestingly, p53-signalling, endocytosis and lysosomal pathways were found to be the most significantly enriched (p≤ 0.05) pathways (Fig. 4A). Moreover, protein-protein interactions of antagomiR-638 de-repressed genes were extracted from the STRING database [http://string-db.org; version 9.1; [13,14]] and revealed a network with TP53 as a central node interacting with a majority of miR-638 targets (Supplementary Fig. S5). This implied that miR-638 might play an important role during melanoma progression by suppressing p53 signalling and lysosomal pathways.

Interestingly, antagomiR-mediated miR-638 inhibition led to dramatically reduced growth of melanoma cells in culture (Fig.4B). Further analysis in human SK-Mel-147 and mouse B16F10 melanoma cell lines (miR-638 negative) indicated significantly higher percentage of apoptotic cells in SK-Mel-147 but not in B16F10 mouse melanoma cells upon miR-638 knockdown indicating high specificity of miR-638-antagomiR (Fig. 4C, D, Supplementary Fig S6A,). Furthermore, knockdown of miR-638 strongly induced p53 expression accompanied with the induction of its downstream apoptosis effectors like p21, Bax and cleavage of pro-caspase 3 (Fig. 4E).

As suggested by the functional enrichment analysis, depletion of miR-638 in melanoma cells also exhibited an increased lysosomal staining as compared with scrambled-control-antagomiR transfected cells, indicating high accumulation of lysosomes and other acidic bodies (Fig. 4F). Interestingly, a number of pro-autophagy genes were found to be upregulated upon mir-638 knockdown (Supplementary Fig S6C). Therefore, we tested whether this increased lysosomal staining was accompanied by enhanced autophagy. Autophagic cells show processing and recruitment of microtubule-associated protein 1 light chain 3 alpha (LC3) and appearance of acidic vesicular organelles (AVOs), which are hallmarks of autophagy. Indeed, knockdown of miR-638 in melanoma cells induced autophagy as evidenced by significantly higher percentage of LC3-EGFP positive cells (Fig. 4G, H; Supplementary Fig. S6D). Interestingly, treatment with rapamycin (a well known mTOR inhibitor and inducer of autophagy) only marginally increased autophagy in miR-638 depleted cells and completely failed to induce autophagy in control melanoma cells (Fig. 4G, H). This suggested that autophagy induced upon miR-638 knockdown is independent of the mTOR pathway.

Taken together, these results clearly demonstrate that miR-638 protects melanoma cells from apoptosis and autophagy by regulating p53 signalling.

### TFAP2A and miR-638 negatively regulate each other

**Figure 5:**
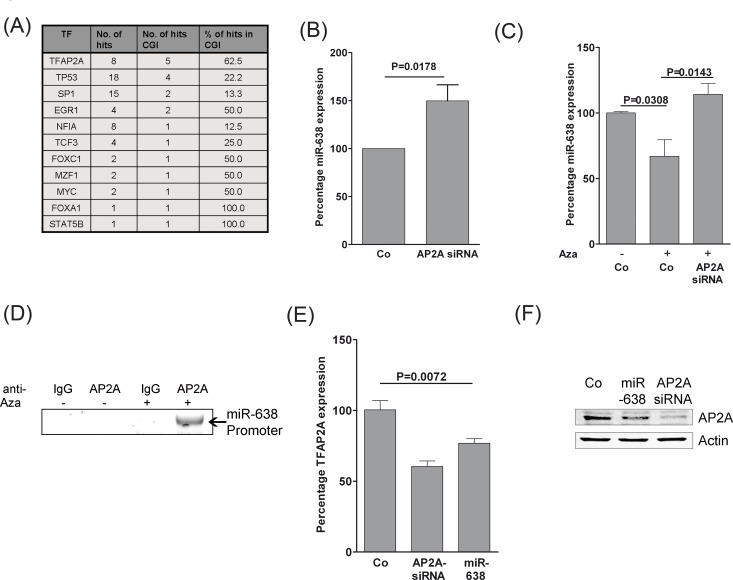
TFAP2A regulates miR-638 in a double-negative feedback mechanism (A) Putative transcription factor binding sites in the 10 kb region upstream of the miR-638 transcription start site were analysed using the MIR@NT@N V1.2.1 framework. The number and percentage of binding sites at methylation-prone CpG islands (CGI) are also included. (B) SK-Mel-147 melanoma cell lines were transfected with scrambled-control (Co) or a *TFAP2A*-specific siRNA (*TFAP2A* siRNA). Expression of miR-638 was measured using TaqMan® qRT-PCR. (C) SK-Mel-147 cells were transfected with the indicated oligomers. One day after transfection, the transfected cells were treated with 3 μM of 5-Aza for 24 h. At this point, complete growth medium was replenished and cells were incubated for another 24 h. Expression of miR-638 was measured using TaqMan® qRT-PCR (mean ± S.E.M.). RNU48 was used as a reference control. (D) Chromatin immunoprecipitation (ChiP) was performed with DMSO or Aza-treated SK-Mel-147 melanoma cell lysates using specific antibodies against AP-2α or isotype-IgG as control. The precipitated DNA was used to amplify a 1 kb sequence upstream of miR-638 transcriptional start site using specific primer pairs. (E) SK-Mel-147 cells were transfected with scrambled-control, *TFAP2A* siRNA or miR-638. Fourty eight hours after transfection, *TFAP2A* mRNA expression was analysed using TaqMan® gene expression assays (mean ± S.E.M.). (F) AP-2α protein levels were analysed using immunoblotting after transfection of SK-Mel-147 cells with the indicated oligomers. β-actin (Actin) was used as the loading control. All biological assays were performed in triplicates and repeated twice upon individual transfections.

In search for transcription factors that might regulate miR-638 expression in melanoma cells, a 10 kb upstream region of the transcription start site of the miR-638 gene was analyzed using MIR@NT@N V1.2.1 transcription factor analysis network [15]. p53, SP1 and AP-2α (encoded by *TFAP2A* gene) were among the predicted transcription factors (Fig. 5A). Surprisingly, a large proportion of AP-2α binding sites (62.5%) was found in methylation-prone CpG islands as compared with p53 (22.2%) and SP1 (13.3%; Fig. 5A).

Next, we tested whether AP-2α (TFAP2A) is able to regulate miR-638 expression. Indeed, knockdown of AP-2α significantly increased the expression of miR-638, suggesting a potential transcriptional repression of miR-638 by AP-2α (Fig. 5B). Further, the effect of demethylation on the expression of miR-638 was analysed. Treatment of melanoma cells with demethylating agent 5-aza-2′-deoxycytidine (5-Aza) resulted in reduction of miR-638 expression, which was reversed upon *TFAP2A* knockdown (Fig. 5C). These findings suggest that miR-638 is indeed regulated by AP-2α and that methylation in AP-2α binding sites negatively interfere with AP-2α activity.

Next, it was determined whether AP-2α directly interacts with miR-638 promoter and if de-methylation can enhance this association. For this purpose, chromatin immunoprecipitation (CHIP) was performed with cell lysates from SK-Mel-147 cells either treated with DMSO or with 5-Aza. Interaction of AP-2α with the miR-638 promoter was found to be more pronounced upon DNA de-methylation which is most likely due to de-methylation of CpG-rich AP-2α binding sites (Fig. 5D). These results strongly argue for a methylation-dependent direct regulation of miR-638 by AP-2α.

We further examined whether *TFAP2A* expression can be regulated by miR-638. *TFAP2A* expression was significantly suppressed by miR-638 both at mRNA and protein levels (Fig. 5E, F). This suggested that *TFAP2A* expression is alteast indirectly regulated by miR-638.

Overall these results suggest that miR-638 and *TFAP2A* regulate each other through a DNA-methylation dependent double negative feedback loop mechanism.

### Epigenetic deregulation of miR-638 promoter confers apoptosis resistance

**Figure 6:**
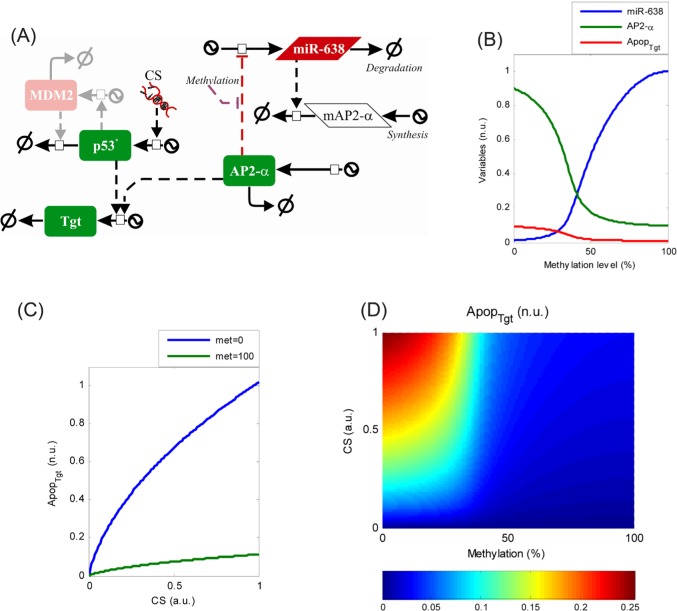
Epigenetic deregulation of miR-638 promoter confers apoptosis resistance (A) A regulatory map describing the miR-638-mediated regulation of apoptosis through its interaction with AP-2α and p53. (B) Model simulation showing the dependency of miR-638 (blue) and AP-2α (green) expression on the methylation of the AP-2α binding site. The simulations suggest an “all-or-nothing” transition in expression levels of miR-638 and AP-2α for intermediate methylation levels. This shift can affect the expression of apoptosis-related genes whose expression is controlled by p53 and AP-2α, here indicated as Apop_Tgt_ (red, scaled 5-fold to facilitate visualization). (C) Simulation representing the expression of pro-apoptotic targets of p53 and AP-2α under increasing levels of cellular stress (CS) for low (blue) and high (green) methylation levels in the AP-2α binding site. (D) Surface plot representing steady-state levels of pro-apoptotic targets determined by varying CS and AP-2α binding site methylation levels.

These experiments suggest that epigenetic deregulation via promoter methylation of miR-638 has the potential to suppress apoptosis at least in part through repression of AP-2α. To better understand the molecular mechanism underlying this regulation, a mathematical model was constructed linking miR-638 deregulation and the emergence of an apoptosis-resistant phenotype. Towards this end, biomedical knowledge about miR-638 and AP-2α was retrieved from publications and databases and integrated into a regulatory map following the approach described recently [16]. More specifically, we searched for transcription factors regulating miR-638 and AP-2α, miR-638 and AP-2α targets, and protein-protein interactions in which AP-2α interacts with proteins involved in apoptosis and cell cycle regulation. We constructed a regulatory map which indicates that the miR-638-mediated regulation of apoptosis involves two interconnected regulatory motifs (Fig. 6A). miR-638 and AP-2α constitute a double-negative feedback loop (AP-2α ⊣ miR-638 and miR-638⊣ AP-2α), a miRNA-involving regulatory motif previously characterized [17]. In addition, AP-2α cooperates with p53 to regulate the expression of cancer genes involved in cell cycle arrest and apoptosis through the creation of nuclear complexes [18,19]

Based on the regulatory map, a kinetic model was constructed using ordinary differential equations which accounts for the temporal evolution of mRNA and protein expression levels for AP-2α and miR-638 and transcriptionally active p53. Finally, an additional variable was introduced accounting for the expression of apoptosis-related genes whose expression is controlled by p53 and AP-2α. The model simulations indicate that the signalling module integrating miR-638 and AP-2α acts as a positive feedback loop. External signals like methylation of the AP-2α binding site in miR-638, even when transient, can trigger a permanent transition from a scenario of permanent AP-2α-mediated miR-638 repression to another of permanent miR-638-induced AP-2α repression (Fig. 6B). Under these conditions and due to the role of AP-2α co-regulating p53 targets, expression of pro-apoptotic genes can be silenced and cancer cells can become resistant to apoptosis initiation (Fig. 6B). Simulations of the model show that this epigenetically-provoked shift in the expression of AP-2α can drastically reduce the expression of p53 pro-apoptotic targets for a wide range of cellular stress levels (*CS*, Fig. 6C). When both regulatory processes are combined, the model suggests that they create a non-linear landscape in the activation of apoptosis (Fig. 6D). These data suggest that cancer cells exhibiting high levels of methylation in the AP-2α binding site of the miR-638 gene promoter are resistant to apoptosis initiation even for high levels of cellular stress, while demethylation of the binding site can sensitize cancer cells to apoptosis.

## DISCUSSION

Over the last decade, strong evidences have been provided for an involvement of miRNAs in facilitating melanoma invasion and metastasis. In the present report we demonstrate that melanoma progression from benign melanocytic lesions to the malignant and metastatic stage is associated with increased expression of miR-638. This suggests that upregulation of miR-638 might be an early event during melanoma initiation and also play an important role during metastasis. Indeed, overexpression of miR-638 enhanced the proliferative, migratory and clonogenic properties of melanoma cells both *in vitro* and metastatic capacities *in vivo.* In line with our findings, miR-638 was shown to suppress BRCA1 and enhance the growth of esophageal squamous cell carcinoma cells [20]. Together, the present study demonstrates the oncogenic role of miR-638 in melanoma.

We identified *TP53INP2* as a direct target of miR-638. Knockdown of miR-638 in melanoma cells increased expression of TP53INP2 and induced significant levels of autophagy. TP53INP2 was shown to be a scaffold protein which participates in autophagosome formation by interacting with pro-autophagy proteins like LC3B and autophagosome transmembrane protein VMP1 [21]. In contrast to a general tumor-suppressive role of autophagy, it is now established that certain tumors, including melanomas, show increased basal autophagy to survive the microenvironmental stress conditions suggesting a pro-tumorigenic role for autophagy [22,23]. However, in the present study miR-638 knockdown-induced autophagy was accompanied by growth suppression and apoptosis. This is in agreement with other studies where treatment of melanoma, glioma or hepatocellular carcinoma cells with metformin, terfenadine or tetrahydrocannabinol (THC) induced autophagy and facilitated cell death [24]. Furthermore, prolonged or extensive autophagy has been shown to induce cell death [25,26]. Therefore, as accumulating evidences suggest that inhibition of autophagy supports tumor formation, strategies exploiting autophagy induction to promote apoptosis might prove to be an effective, new therapeutic approach.

Interestingly, *TP53INP2* shares 30% sequence identity with *TP53INP1*, which plays an important role in tumor suppression by direct interaction with p53 [27,28]. Thus we hypothesized that TP53INP2 protein might also act as a tumor suppressor. Indeed, overexpression of *TP53INP2* cDNA strongly attenuated growth and invasion of melanoma cells. This suggests that pro-tumorigenic and metastatic effects of miR-638 may be largely mediated by suppression of *TP53INP2*.

The presence of wild type p53 implicates the involvement of other factors mediating suppression of this major tumor suppressor pathway in melanoma. MDM2 and iASPP/PPP1R13L (protein phosphatase 1, regulatory subunit 13 like) suppress p53 pro-apoptotic signalling and thereby promote melanoma progression [29]. Inhibition of MDM2 and iASPP phosphorylation reinstated p53 levels and induced growth suppression and apoptosis in melanoma cells [29]. Interestingly, in the present study, functional enrichment analysis revealed p53 signalling to be significantly suppressed by miR-638. Knockdown of endogenous miR-638 led to apoptosis likely due to induction of p53 and its downstream genes p21 and Bax. This suggests that miR-638 inhibits p53 pro-apoptotic signalling as a prerequisite for melanoma development and progression and therefore might serve as a potential therapeutic target in melanoma.

In search for transcription factors, which might regulate the expression of miR-638, a direct interaction and suppression of miR-638 by AP-2α was identified. It is well-understood that melanoma progression is associated with loss of expression of AP-2 proteins, and this correlates with poor prognosis and advanced stages of the disease [30,31]. Loss or deletions of genomic loci encoding for *TFAP2* family members were found in some, but not all, melanomas, suggesting involvement of other mechanisms for control of gene expression [32]. It is known that elimination of TFAP2A from non-metastatic primary melanoma cells increases their malignancy while re-expression abrogates it, by controlling transcription of genes such as *MCAM-MUC18*, *MMP2*, *PAR1*, *VEGF*, *BCL2*, *CDKN1A*/p21, *E-cadherin* and *KIT* [33-35]. In line with these data, overexpression of miR-638 during melanoma progression may be explained, at least in part, by loss of AP-2α. Interestingly, the AP-2α binding sequences in the miR-638 promoter were found to be prone to methylation at CpG islands. Treatment of melanoma cells with DNA demethylating agent 5-Aza reduced miR-638 expression and increased the interaction between AP-2α and the miR-638 promoter. This suggests that miR-638 expression is repressed by AP-2α, and methylation-mediated epigenetic silencing of AP-2α binding sequences at miR-638 promoter contributes to aberrant expression of miR-638 during melanoma progression.

From a therapeutic perspective, this study strongly argues towards a combinatorial treatment involving epigenetic mechanisms and conventional and/or innovative approaches using small molecule inhibitors.

## MATERIALS AND METHODS

### Melanoma tissues and cell lines

For miRNA expression profiling cryopreserved microdissected patient material from 7 primary melanomas, 9 lymph node metastases, and 8 distant cutaneous melanoma metastases, respectively, was used. Analyses of patient material were done after informed consent of patients and approval by the local Ethics Committee (AZ 08-220). SK-Mel-cell lines were kindly provided by M. Soengas, Department of Dermatology, University of Michigan, Ann Arbor, MI, USA. Melanoma cell lines BRO, A-375, HT144, RPM-MC and 1F6, were obtained from U. Anderegg, Department of Dermatology, Venereology and Allergology, University of Leipzig. Freshly isolated normal human epidermal melanocyte pellets were purchased from PromoCell, Heidelberg, Germany (C-14043). The cell lines were regularly tested for mycoplasmal contamination.

### Microarray analyses

Whole genome cDNA microarray (Illumina Human HT-12 v4 Expression BeadChip Kit, San Diego, CA 92122 USA) analyses were performed in duplicates using RNA extracted from mock transfected SK-Mel-147 cells or transfected with a miR-638 or antagomiR-638. The data was processed and analyzed using the Bioconductor package lumi [36] in the statistical programming environment R.

### MicroRNA expression profiling

TaqMan® low-density arrays (TLDA; human microRNA Cards A v2.1 & B v2.0, Applied Biosystems, Darmstadt, Germany) were used for measuring the expression of 667 human miRNAs from miRBase version v.10 with an Applied Biosystems 7900HT. Raw data were exported using SDS Relative Quantification Software version 2.2.2 (Applied Biosystems) with automatic baseline and threshold settings.

### Small interfering (si)RNA transfection

siRNA and miRNA transfections of the melanoma cells were performed as previously described [37].

### Cell cycle analysis

Cell cycle assays were performed as previously described [37]. The cell cycle distribution was analyzed using BD-FACSCalibur (BD Biosciences Pharmingen, San Diego, CA, USA).

### Proliferation Assay

Proliferation assays were performed as previously described [37]. The UV absorption was measured 2 h after addition of XTT reagent at 492 nm using a Biotek Synergy^TM^ HT microplate reader (BioTek Instruments, Inc, Vermont, USA).

### Wound-healing assays

Wound healing assays were performed with SK-Mel-147 (2.5 × 10^4^ cells/well) and SK-Mel-28 (5 × 10^4^ cells/well) cells, respectively, using Oris^TM^ Cell Migration Assay (CMA5.101) according to the manufacturer's instructions, after transfection of cells with the indicated oligonucleotides. Melanoma cells were treated with 10 μM mitomycin C for 1 h to inhibit proliferation. The images of the wound area were captured microscopically at different time points using a BZ-9000 fluorescence microscope and analyzed using a *BZ-II analyzer* (Keyence, Neu-Isenburg, Germany).

### Invasion assays

Assays were performed using Boyden chamber inserts coated with matrigel layer over a 8 μm porous membrane (24 well Thincert^TM^, Greiner Bio-One, Frickenhausen, Germany The invaded cells stained with DAPI. Microscopic pictures were acquired at 10 X magnifications using a BZ-9000 fluorescence microscope and analyzed using a *BZ-II analyzer* (Keyence, Neu-Isenburg, Germany).

### Colony-forming assay

Colony forming assays were performed as previously described [37].

### Soft agar assay

Six-well-plates were coated with a 1% agarose layer and incubated for 1 h to solidify. SK-Mel-28 melanoma cells were trypsinized and counted. Appropriate numbers of cells were resuspended in prewarmed (37°C) soft agar solution containing 0.7% agarose and 2x DMEM containing 20% FCS (1:1). Melanoma cells (2 × 10^4^) were then seeded per well of a 6-well-plate in soft agar, and 1 h after seeding, the cells were covered with 2X DMEM containing complete growth medium. The cells were incubated for 15-20 days with change in growth medium twice a week. After this, the cells were fixed with 4% paraformaldehyde solution and stained with 0.5% crystal violet and analysed for growing clones using a BZ-9000 microscope (Keyence).

### Immunoblotting

Immunoblotting was performed as described [37]

### Luciferase reporter assay

Luciferase reporter gene assays were performed as described [37]. In brief, *TP53INP2* 3′-UTRs were cloned into the pmirGLO vector. The firefly and renilla luciferase reading were obtained using Dual-Luciferase Reporter Assay System (Promega GmbH, Mannheim, Germany).

### Apoptosis assay

Antagomir-638-transfected SK-Mel-147 human melanoma cells and B16F10 mouse melanoma cells were trypsinized, washed and incubated with an annexin V antibody diluted in binding buffer (1:20) for 30 to 45 min. After this, propidium iodide (PI) solution (diluted in the binding buffer at 1:150) was added to the annexin V stained cells. Shortly after addition of PI solution, the percentage of apoptotic and secondary necrotic cells was determined using a BD FACSCanto II system at the indicated time points. Analysis was performed using the BD FACSDiva Software (BD Biosciences, San Jose, CA, USA).

### Immunofluorescence staining

For immunofluorescence analyses of SK-Mel-147 cells, cells were plated on glass coverslips in 24-well-plates and transfected with 100 nM antagomiR-638 or a control miRNA. At indicated time points, cells were stained with lysotracker red (Invitrogen-Molecular Probes, Eugene, Oregon, USA) for 30 min, after which they were fixed in 4% formaldehyde for 1 h at room temperature. Counterstaining was performed with DAPI for visualization of nuclei. Autophagy analysis was performed with EGFP-LC3B overexpressing SK-Mel-28 and SK-Mel-147 melanoma cells. The percentage of EGFP-LC3B transfected melanoma cells was determined by their LC3B-GFP positivity. Fluorescence intensities were measured in at least 5 viewing areas for 50 cells per section. Microscopic analysis was performed with a BZ-9000 fluorescence microscope and analyzed using a *BZ-II analyzer* (Keyence).

### Chromatin immunoprecipitation

Chromatin Immunoprecipitation (ChIP) was performed with cell lysates prepared from DMSO or 5-Aza-treated SK-Mel-147 melanoma using EZ-ChIP^TM^ chromatin immunoprecipitation kit (Millipore, Darmstadt, Germany) according to the manufacturer's instructions.

### Lentiviral transduction

SK-Mel-147 melanoma cells stably expressing a scrambled-control or miR-638 were produced using HMR 5872 and shMIMIC lentiviral microRNA viral particles, respectively, according to the manufacturer's specifications (Thermo Scientific, Lafayette, CO, U.S.A.). Positively transduced cells were double-selected for puromycin (1 μg/ml) resistance and green fluorescence protein (GFP) expression. This medium was replaced by normal culture medium 24 h before start of experiments.

### Animal experiments

SK-Mel-147 human melanoma cells overexpressing miR-638 or scrambled-control were trypsinized and single cells were enriched by filtering them through a cell strainer. Melanoma cells (2 × 10^6^) were then injected into the lateral tail vein of NSG (NOD *scid* IL2 receptor gamma chain knockout) mice [38]. Tissue sections (8 μm thickness) were stained with hematoxylin and eosin (H&E). The images were acquired using BZ-9000E microscopic system (Keyence) at 4-fold magnification, and the extent of metastasis was analysed with BZ-II analysis software (Keyence). All animal experiments were performed according to the institutional and state guidelines, and the committee of animal welfare of Saxony approved animal protocols used in this study (TVV 53/11).

### Statistical analyses

Statistical significance of differential expressions were analysed by unpaired *t*-test as indicated in the figure legends. Statistical evaluation for proliferation, migration and invasion assays was performed by means of analysis of variance (ANOVA); Benjamini and Hochberg test was used for pairwise comparisons. *P* values ≤ 0.05 were regarded as statistically significant.

## SUPPLEMENTARY MATERIAL FIGURES AND TABLES


