# Supplementary material for: miR-638 promotes melanoma metastasis and protects melanoma cells from apoptosis and autophagy
Source: Oncotarget. 2014 Dec 26;6(5):2966–80. doi: 10.18632/oncotarget.3070 (PMC4413631; doi:10.18632/oncotarget.3070)
Supplement: Supplementary file 1 [file oncotarget-06-2966-s001.pdf]

miR-638 promotes melanoma metastasis and protects melanoma cells from apoptosis and autophagy

Supplementary Material

Supplementary Figures

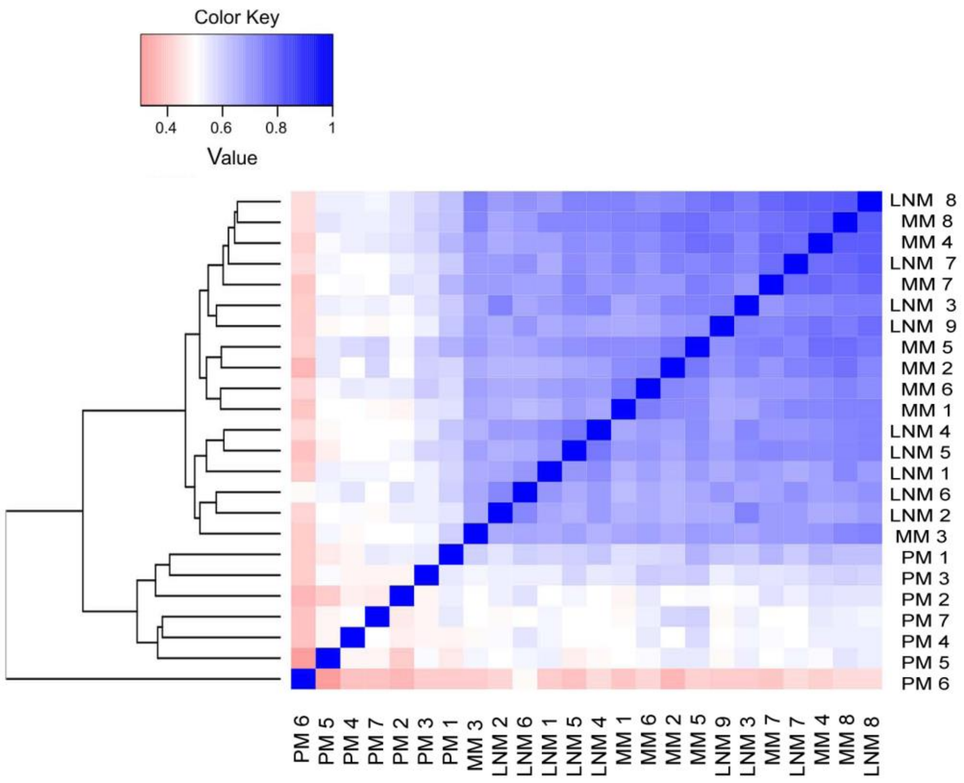

Supplementary Figure S1: Metastatic lesions show high levels of correlation for miRNA expression profiles. Heatmap of sample correlation (Pearson's correlation) based on normalized Ct values. Correlation coefficients of miRNA expression profiles of samples pairs range between 0 = no correlation and 1 = identical, represented in the given color scheme. Note that miRNA patterns for samples of primary melanomas (PM) do not correlate well. Samples of lymph node (LNM) and distant cutaneous metastases (MM) show higher levels of correlation and were clearly separated from primary tumors.

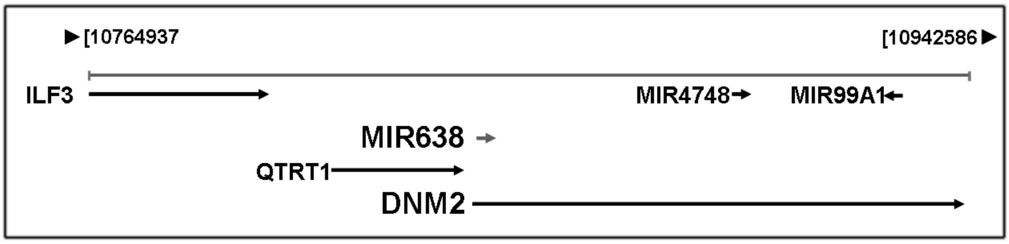

Supplementary Figure S2: Gene locus for miR-638 and its host gene DNM2 on chromosome 19p13.2.

Supporting Information Fig 3

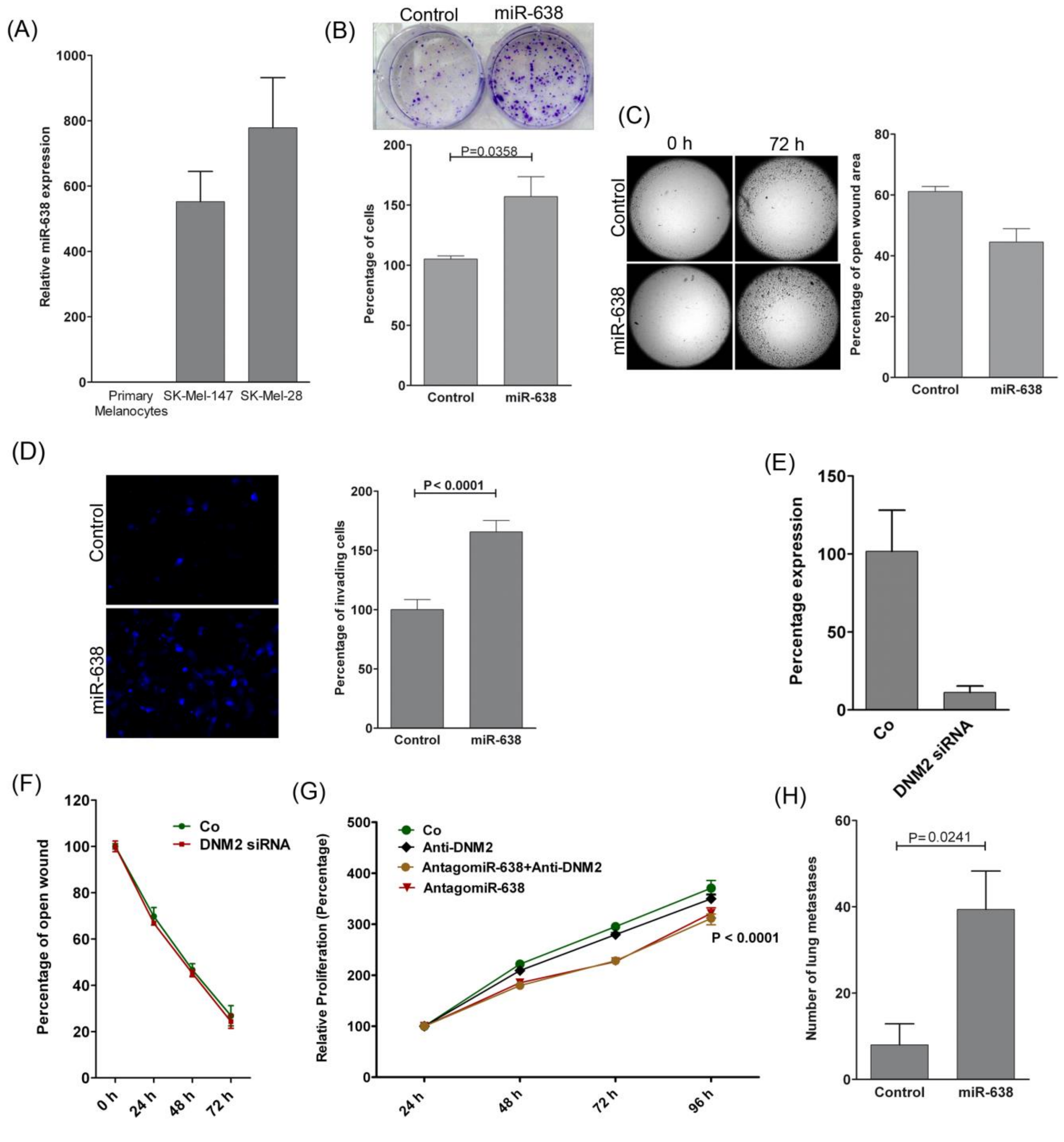

Supplementary Figure S3: miR-638 promotes tumorigenic and metastatic properties of melanoma cells *in vitro* (A) Endogenous miR-638 expression in the indicated cells. All miRNA expression analyses were performed using TaqMan® qRT-PCR technology. RNU48 expression was used as universal reference control for miRNAs. Data are given as mean ± S.E.M. of the relative miR-638 expression. **(B)** Colony-forming assay using SK-Mel-28 cells overexpressing a non-targeting control miRNA or miR-638. Data are given as mean ± S.E.M. of the average colony number. **(C)** Migration assays were performed for SK-Mel-28 cells overexpressing a non-targeting control miRNA or miR-638. Microscopic pictures were taken at indicated time points. Graphs represent summaries of three independent experiments. Data

are given as mean ± S.E.M for the percentage of the remaining open wound area as compared with 0 h time point. **(D)** Matrigel invasion assays were performed for SK-Mel-147 cells transiently overexpressing a control plasmid or *TP53INP2* cDNA co-transfected with a control miRNA or miR-638. Microscopic pictures were taken at 48 h (mean ± S.E.M, n=3). **(E)** SK-Mel-28 melanoma cells were transfected with *DNM2*-specific siRNA or non-targeting control oligomers. After 48 h of transfection, mRNA expression of *DNM2* was analysed using TaqMan® gene expression assays. The *DNM2* expression data are given as mean ± S.E.M percentage of relative expression. **(F)** Migration assays were performed with DNM2-depleted SK-Mel-28 cells. Microscopic pictures were taken at indicated time points (0, 24, 48 and 72 h). Graphs represent summaries of three independent experiments, with data given as mean ± S.E.M for the percentage of the remaining open wound as compared with 0 h time point. **(G)** XTT cell proliferation assays were performed after transfection of SK-Mel-28 cells with indicated oligomers. UV absorption was measured at 24, 48, 72 and 96 h. Data are expressed as percentage of relative proliferation compared with controls at 24 h time point (mean ± S.E.M). **(H)** Number of lung metastasis were counted and analysed in the H&E stained lung sections. A minimum of 3 lung sections per mouse were quantitated (mean ± S.E.M.). All biological assays were performed in triplicates and repeated twice upon individual transfections and assay measurement.

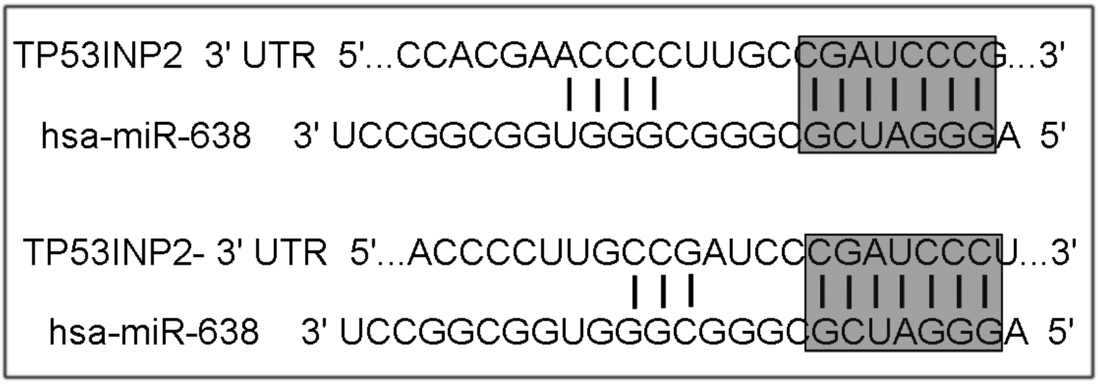

Supplementary Figure S4: TP53INP2 is a conserved target for miR-638. TP53INP2 has conserved miR-638 seed binding sites in their 3′-UTR region as shown by highlighted sequence alignments; derived from http://www.targetscan.org.

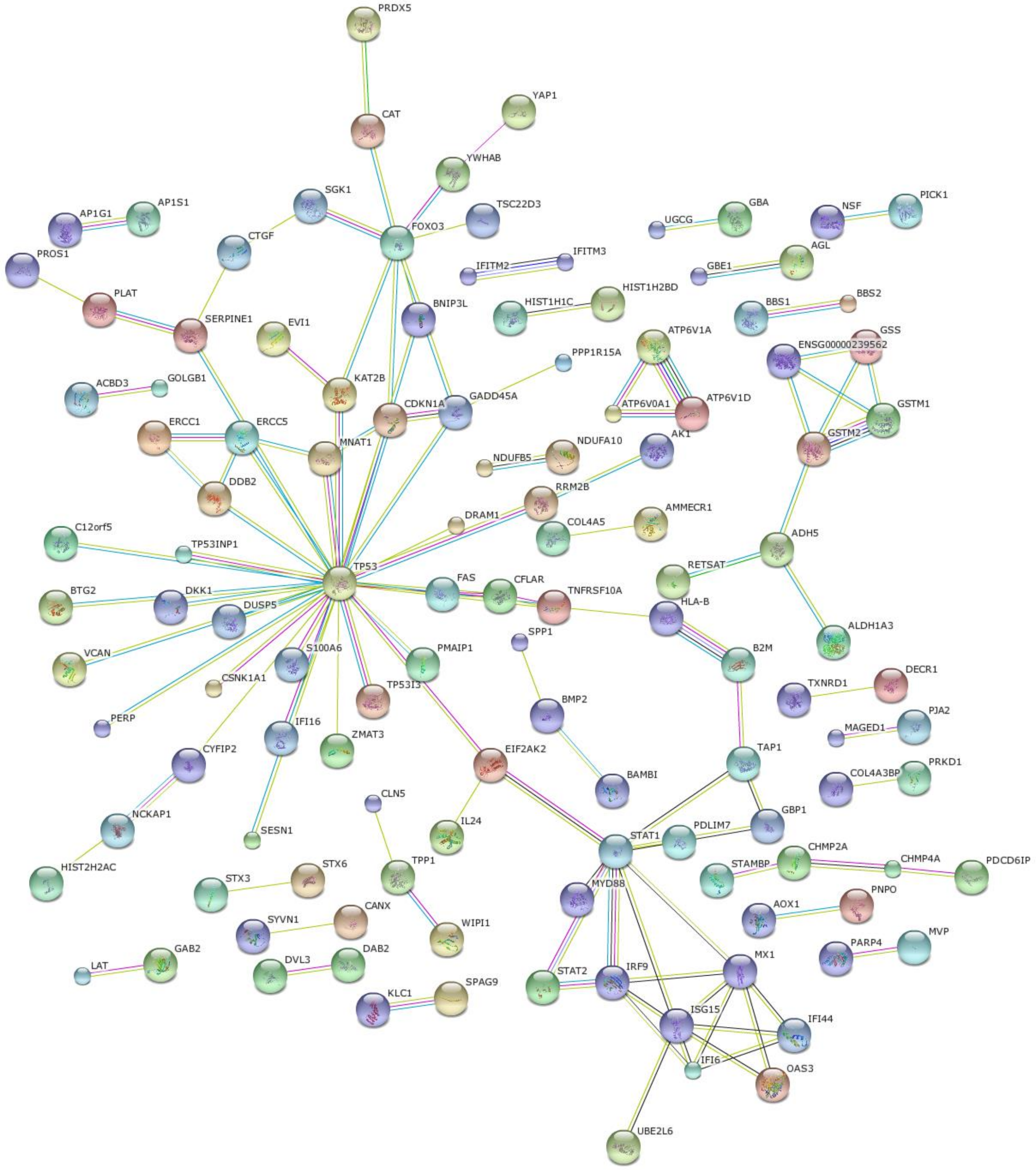

Supplementary Figure S5: miR-638 represses TP53 gene network and its targets. Protein-protein interactions of antagomiR-638 de-repressed genes were extracted from the STRING database [http://string-db.org; version 9.1; (Franceschini et al., 2013)]. The STRING gene/protein interaction network shows interactions with TP53 as a central node interacting with majority of miR-638 targets.

Supporting Information Fig 6

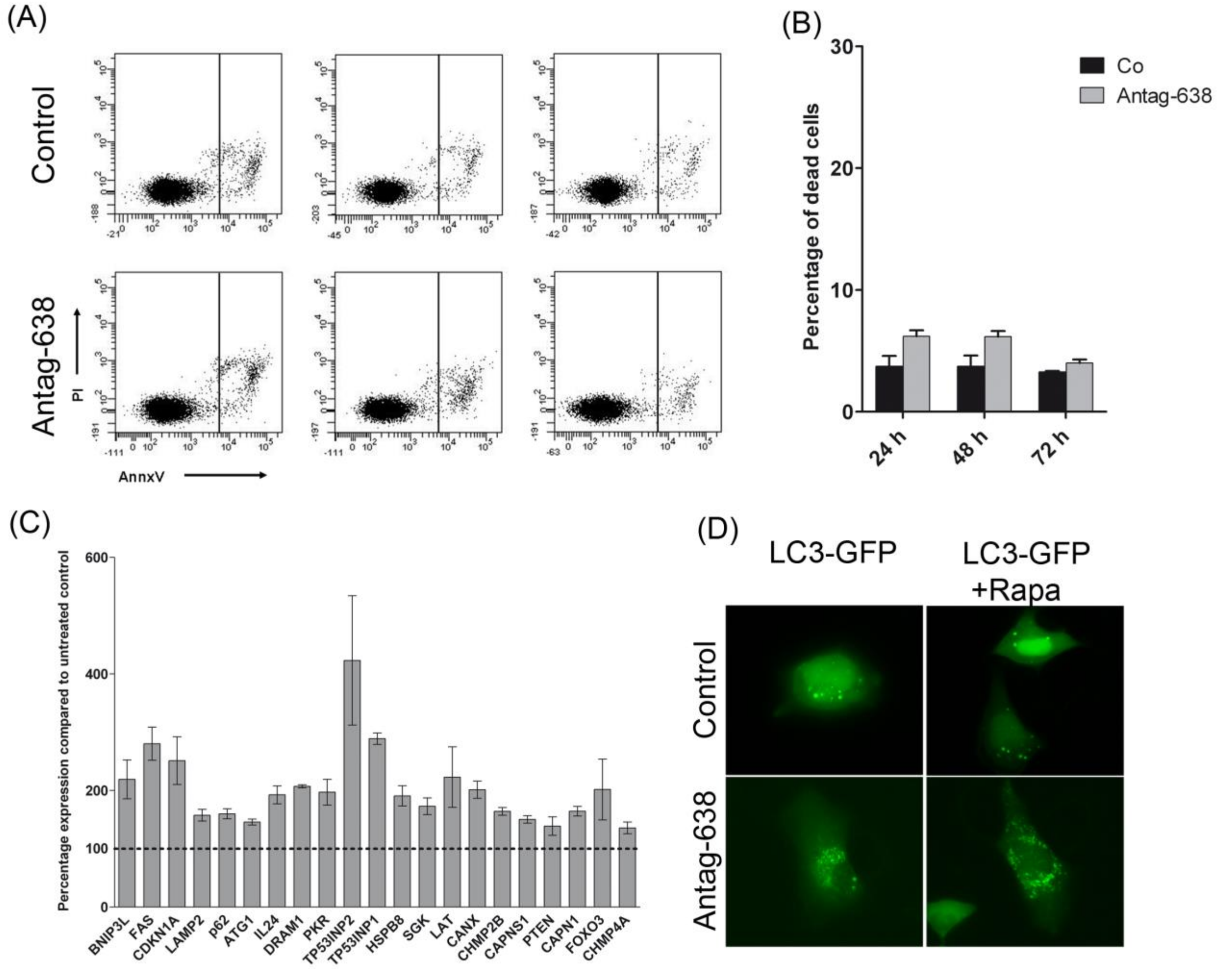

Supplementary Figure S6: Knockdown of miR-638 induces apoptosis and autophagy in melanoma cells in vitro (A) B16-F10 mouse melanoma cells were transfected with non-targeting control (Co), miRNA or antagomiR-638 (Antag-638). At indicated time points, cells were stained for annexin-V/propidium iodide (PI) and apoptotic cells were analysed by flow cytometry. **(B)** Graphs indicate percentage of dead cells (mean ± S.E.M). Apoptosis assays were performed in triplicates and repeated twice upon individual transfections and assay measurement. **(C)** List of autophagy related genes upregulated in response to miR-638 knockdown (mean ± S.E.M) **(D)** SK-Mel-147 melanoma cells were co-transfected with an EGFP-LC3B construct and a non-targeting control miRNA or antagomiR-638 (Antag-638). After 24 h of transfection, the cells were either left untreated or treated with 100 nM rapamycin for another 24 h, after which they were fixed and microscopic images were taken (x60).

Supplementary Tables

Supplementary Table S1: Differentially expressed miRNAs at different stages of melanoma progression

	**miRNA name** (official name)	**MM vs PM**		**MM vs LNM**		**LNM vs PM**	
		**adj. p- value**	**FC**	**adj. p- value**	**FC**	**adj. p-value**	**FC**
	hsa-miR-126* (hsa-miR-126-5p)	0.0004	76.7719			0.0000	75.0130
	hsa-miR-638	0.0103	8.2213			0.0009	21.9030
	hsa-miR-30e* (hsa-miR-30e-3p)	0.0103	6.9208			0.0006	11.8506
	hsa-miR-374b	0.0093	12.2426			0.0378	6.1316
	hsa-miR-454	0.0474	6.3270			0.0076	9.3536
	hsa-miR-30e (hsa-miR-30e-5p)	0.0040	6.8688			0.0006	8.7370
	hsa-miR-151-3p	0.0014	7.4725			0.0000	7.7017
	hsa-miR-378	0.0211	7.9801			0.0340	7.0288
	hsa-miR-923	0.0021	5.0905			0.0002	8.1688
	hsa-miR-628-5p	0.0016	6.5895			0.0002	5.5144
	hsa-miR-7-1* (hsa-miR-7-1-3p)	0.0093	4.9722			0.0006	6.3135
	hsa-miR-335*	0.0499	6.6154			0.0076	4.1487
	hsa-miR-590-5p	0.0021	5.0401			0.0158	4.5644
	hsa-miR-186	0.0070	4.1927			0.0020	4.7558
	hsa-miR-30d	0.0429	4.1768			0.0388	3.8147
	hsa-miR-101	0.0429	3.7619			0.0285	4.0331
	hsa-miR-93	0.0234	0.2479			0.0388	0.2070
	hsa-miR-193b	0.0061	0.1243			0.0010	0.0714
	hsa-miR-199a-3p			0.0178	5.0575	0.0618	0.1104

PM, primary melanomas; LNM, lymph node metastases; MM, distant cutaneous metastases FC, fold change.

Supplementary Table S2: List of primary melanoma specimens analyzed by TaqMan® real-time PCR for miRNA expression post microdissection

Number	Subtype *)	Vertical tumor thickness (mm)	Clark Level
1	NMM	1.5	IV
2	SSM	2.4	IV
3	SSM	8.0	V
4	NMM	5.3	IV
5	SSM	1.0	III
6	SM	1.1	III
7	SSM	1.5	III
8	SSM	1.9	IV
9	NMM	3.0	IV
10	NMM	5.4	IV
11	NMM	1.7	III
12	SSM	2.8	III
13	NMM	4.4	V
14	NMM	1.1	III
15	SSM	1.0	III
16	NMM	2.1	IV
17	NMM	8.6	IV
18	NMM	4.8	III
19	NMM	3.5	III
20	SSM	2.5	III
21	SSM	1.0	III
22	NMM	5.0	III
23	SCM	8.0	IV
24	NMM	4.1	III
25	SSM	1.3	III
26	NMM	2.3	IV
27	SSM	1.7	III
28	SSM	1.1	III
29	ALM	1.7	III
30	NMM	3.2	III
31	SSM	1.6	IV
32	SSM	1.5	III
33	ALM	1.3	III
34	SSM	1.2	III
35	SSM	1.7	III-IV

*) NMM, nodular malignant melanoma; SSM, superficial spreading malignant melanoma; ALM, acro-lentiginous malignant melanoma; SCM Spindle cell melanoma

Supplementary Table S3: Putative targets of miR-638 mediated post-transcriptional regulation.

Target genes	Log_{10} FC_{total}	FC (miR-638 vs NT)	FC (antagomiR vs NT)	predicted by # algorithms
ZNF185	2.5391	-38.371	307.676	5
TP53INP2	2.8513	-56.203	654.009	5
ODZ4	2.4666	-193.07	99.792	5
DUSP3	2.2810	-71.301	119.715	5
DSCR3	2.1001	-59.207	66.718	5
COMMD1	2.3851	-30.584	212.168	5
TRAM2	2.4903	-148.41	160.876	4
APCDD1L	2.2982	-2.022	196.702	4
PRKAR1A	2.8749	-455.749	294.11	4
UBL3	2.2761	-62.874	126.011	4
BTG2	2.3763	-49.897	187.999	4
CIAO1	2.3472	-41.596	180.868	4
ARHGAP1	2.2692	-127.718	58.15	4
C7orf50	2.2097	-2.294	159.782	4
COMMD6	2.7447	-445.029	110.613	4
KIAA0174	2.1327	-75.93	59.838	4
SLC2A1	2.5544	-345.397	13.047	4
VPS41	2.4887	-89.573	218.598	4
INTS3	2.3658	-115.737	116.447	3
C5orf15	2.7343	-404.579	137.836	3
ZBTB4	2.7003	-206.187	295.39	3
ITGA3	2.8622	-387.605	340.537	3
SLC35B2	2.0350	-90.974	17.437	3
CD59	2.7929	-199.609	421.183	3
AK2	2.2387	-111.979	61.285	3
CREB3L2	2.2562	-108.867	71.547	3
MKRN1	2.4673	-132.41	160.945	3
TRAK2	2.0584	-78.524	35.887	3
SIDT2	2.2289	-20.304	149.096	3
PARP3	2.4063	-43.468	211.401	3

Genes that were significantly differentially expressed ($p \leq 0.01$) and miR-638 inversely correlated in Illumina BeadChip arrays of miR-638, antagomiR-638, and control-transfected

SK-Mel-147 cells, with a total $\log_{10}$ fold change of 2 or more (sum of absolute fold changes $|FC_{miR-638}|$ and $|FC_{antagomiR-638}|$), were compared with predicted miRNA-638 targets from eight target prediction algorithms [source: miRWalk database; (Dweep et al., 2011)]. The table lists only those targets that were predicted by at least three different algorithms.

Supplementary Materials and Methods

MicroRNA expression profiling

RNA extracted from microdissected sections of PM, LNM and macrodissected sections of MM patient samples were used to avoid stromal cell contamination. Total RNA was extracted using the *mir*Vana microRNA extraction kit (Ambion, Life Technologies, Darmstadt, Germany). Total RNA (1 µg) was reverse transcribed using the High Capacity cDNA reverse transcription kit (Applied Biosystems) and TaqMan® Micro-RNA Megaplex RT Human Pool Sets A & B. The arrays were performed according to the manufacturer's instructions with an Applied Biosystems 7900HT thermocycler under the following conditions: 50°C for 2 min, 94.5^{o}C for 10 min, followed by 40 cycles of 97°C for 30 sec and 59.7^{o}C for 1 min. Raw data were exported using SDS Relative Quantification Software version 2.2.2 (Applied Biosystems) with automatic baseline and threshold settings.

Data analysis for microRNA expression profiling

The raw *Ct* (threshold cycle) values from the 7900HT Fast Real-Time PCR System were processed and analyzed using the Bio conductor package HTqPCR Version 1.12.0 [1] in the statistical programming environment R. Rank invariant controls were chosen for ΔCt normalization. For the A plates of all samples the arithmetic mean *Ct* values of MammU6, RNU44, RNU48 were used for normalization, while in B plates the controls MammU6, RNU44, RNU48, RNU24, RNU43 and RNU6B (last three not present on plate A design) were used for normalization. For the relative quantification of miRNA expression a cut-off was set at a *Ct* value of 40. Probes with *Ct* values of 40 or greater were considered as undetermined. Sample correlation (Pearson's correlation) was assessed based on normalized *Ct* values in a heatmap using hierarchical clustering (Supplementary Fig. 1).

Differentially expressed miRNAs between disease state pairs (PM, LNM, MM) were determined by Student's *t*-tests. The results were corrected for multiple testing by the Benjamini and Hochberg method to control the false discovery rate. The miRNAs, for which half or more of the biological replicates were undetermined (e.g., ≥40 *Ct*) in one condition, were discarded from the results. miRNAs with an adjusted p-value ≤ 0.05 and an absolute fold change |FC| > 3 were considered for further analysis. Supplementary Table 1 lists distinctively regulated miRNAs, i.e., those that are differentially expressed in one state as compared to the other two states are listed.

Microarray analyses

Whole genome cDNA microarray (Illumina Human HT-12 v4 Expression BeadChip Kit, San Diego, CA 92122 USA) analyses were performed in duplicates using RNA extracted from mock transfected SK-Mel-147 cells or transfected with a miR-638 or antagomiR-638. ANOVA was performed on the quantile normalized data of the distinctively treated cells. Differentially expressed genes were considered significant when adjusted $p \leq 0.01$ (corrected for multiple testing by the Benjamini and Hochberg method). The genes which were both exclusively suppressed by miR-638 overexpression and de-repressed by miR-638 knockdown were considered for further validation. Finally, we reduced the number of candidates by considering only those genes with a total $\log_{10}$ fold change of 2 or more (sum of absolute fold changes). The data was processed and analyzed using the Bioconductor package lumi [2] in the statistical programming environment R.

Small interfering (si)RNA transfection

Briefly, SK-Mel-28 or SK-Mel-147 cells were seeded in either 24-well or 6-well plates. 40 nM siRNA (DNM2 siRNA #s4213, *TP53INP2* siRNA #s14004, TFAP2A siRNA #s226936, scrambled siRNA #D-001810-10-05) or 100 nM mature miRNA mimic miR-638 #C-300965-01-0005, miRIDIAN microRNA Hairpin Inhibitor for miR-638 (antagomiR-638) #IH-300965-03-0005, or mimic negative control miRNA (#1-CN-001000-01-05) or miRIDIAN microRNA Hairpin Inhibitor Negative Control (control-antagomiR) #IN-001005-01, all from Dharmacon RNAi Technologies (Thermo Fisher Scientific, Lafayette, CO, U.S.A.), were transfected using lipofectamine RNAimax reagent (Invitrogen, Karlsruhe, Germany).

Cell cycle analysis

Cell cycle assays were performed as previously described [3]. Briefly, SK-Mel-147 or SK-Mel-28 cells, were transfected with indicated oligonucleotides. Twenty four hours after transfection the cells were fixed with 70% ethanol. Ethanol-fixed cells were centrifuged at 3000 rpm for 5 min, washed twice with PBS and then incubated with 0.5 ml PBS containing 0.1 mg/ml RNase A and 50 µg/ml propidium iodide (PI) for 30 min at room temperature. The cell cycle distribution was analyzed using BD-FACSCalibur (BD Biosciences Pharmingen, San Diego, CA, USA).

Proliferation Assay

Twenty four hours after transfection of SK-Mel-147 or SK-Mel-28 cells with the indicated oligonucleotides or cDNA plasmids, cells were seeded in a 96-well-plate (4×10^3 cells/well). Proliferation was analyzed at given time points using the Cell Proliferation Kit II (Roche Molecular Biochemicals, Mannheim, Germany). The UV absorption was measured 2 h after addition of XTT reagent at 492 nm using a Biotek Synergy™ HT microplate reader (BioTek Instruments, Inc, Vermont, USA).

Invasion assays- Assays were performed using Boyden chamber inserts coated with matrigel layer over a 8 µm porous membrane (24 well Thincert™ , Greiner Bio-One, Frickenhausen, Germany). In brief, SK-Mel-147 cells (5 x 10^3) suspended in serum-free culture medium were seeded in the matrigel insert. In the lower chamber culture medium contained 20% fetal calf serum. Two to 4 h after seeding, the cells were treated with 10 µM mitomycin C for 1 h to inhibit proliferation. After 48 h, the inserts were removed, washed with PBS and fixed with 4% formaldehyde. The cells remaining on the inner membrane were removed and the invaded cells stained with DAPI. Microscopic pictures were acquired at 10 X magnifications using a BZ-9000 fluorescence microscope and analyzed using a BZ-II analyzer (Keyence, Neu-Isenburg, Germany).

Colony-forming assay

SK-Mel-28-control or SK-Mel-147 cells transfected with the indicated oligonucleotides were seeded at low density (500 cells/well) in a 6-well-plate and allowed to grow for 10-12 days. The cells were then stained with 0.1% crystal violet and analyzed microscopically. Quantification was performed by counting the total number of colonies in each well.

Immunoblotting

Immunoblotting was performed as described [3]. The following primary antibodies against p53 (sc-5285), p21 (sc-7989), Bax (sc-493), pan-Caspase-3 (sc-56055), and AP-2α (sc-12726) were all purchased from Santa Cruz Biotechnologies (Santa Cruz, CA, U.S.A.). Anti-TP53INP2 (AP17800PU-N) was purchased from Acris GmBH (Herford, Germany) and. Anti-β-actin mouse monoclonal Ab (8H10D10, Cell Signaling Technology, MA, USA) was used to verify equal loading of proteins. The following secondary antibodies were used for immunodetection (all from LI-COR Biosciences, Bad Homburg, Germany): IRDye 680LT goat anti-rabbitIgG (926-68021), IRDye 800CW donkey anti-mouseIgG (926-32212). Immunodetection and quantification of protein bands was performed with a LI-COR Odyssey scanner and Odyssey 3.0 software (LI-COR Biosciences).

Luciferase reporter assay

Luciferase reporter gene assays were performed as described [3]. In brief, *TP53INP2* 3'-UTRs were cloned into the pmirGLO vector. The miR-638 binding seed regions were mutated with mutant primers (mentioned below) using a site-directed mutagenesis kit (QuickChange Lightning Site-Directed Mutagenesis Kit, Agilent Technologies, La Jolla, CA, U.S.A.). Mutations at miR-638 binding sites were confirmed by DNA sequencing. The wild type or mutated (single or double) *TP53INP2* 3'-UTR constructs were co-transfected with miR-638 mimics into SK-Mel-147 cells. After 48 h, luciferase activity was measured using the Dual-Luciferase Reporter Assay System (Promega GmbH, Mannheim, Germany). The firefly luciferase readings were normalized against the renilla luciferase values.

Tumor protein 53 inducible protein 2 (*TP53INP2*)

1) Single-Mut-FP 5'-GGC CGC GCC ACG AAC CCC TTG CCa gTg gCG ATC CCT GTC GGG CTC CTC CG-3'

2) Single-Mut-RP 5'-CG GAG GAG CCC GAC AGG GAT CGc cAc tGG CAA GGG GTT CGT GGC GCG GCC-3'

3) Double Mut-FP 5'-GGC CGC GCC ACG AAC CCC TTG CCa gTg gCa gTg gCT GTC GGG CTC CTC CG-3'

4) Double Mut-RP 5'-CG GAG GAG CCC GAC AGc cAc tGc cAc tGG CAA GGG GTT CGT GGC GCG GCC-3'

Chromatin immunoprecipitation

Chromatin Immunoprecipitation (ChIP) was performed with cell lysates prepared from DMSO or 5-Aza-treated SK-Mel-147 melanoma using EZ-ChIP™ chromatin immunoprecipitation kit (Millipore, Darmstadt, Germany) according to the manufacturer's instructions. In brief, 5 x 10^6 SK-Mel-147 melanoma cells were trypsinized and fixed with 1% formaldehyde. The fixed cells were lysed using SDS lysis buffer containing 1x protease inhibitors. Lysates were then sonicated to obtain DNA fragments ranging from 200-1000 bp. The insoluble fraction was removed by centrifugation at 10,000 g at 4°C for 10 min. Ten micrograms of monoclonal AP-2α antibody or isotype IgG were incubated with magnetic Protein G beads (Dyna beads, Life Technologies, Darmstadt, Germany) at 4°C overnight with rotation. After washing, the antibody-coated beads were incubated with the supernatant containing genomic DNA fragments for 1 h at 4°C with rotation. After washing, the protein-DNA complexes were eluted from the beads using the elution buffer according to manufacturer's instructions. The beads were magnetically separated from the eluate containing the protein-DNA complexes, which were de-crosslinked using 5 M NaCl. After RNAse A and proteinase K treatment, the DNA was purified from the eluate using DNA binding spin columns. Two microliter of the DNA purified from AP-2α-specific or IgG control pulldown fractions were used to amplify a 1000 bp promoter region upstream of miR-638. The PCR conditions consisted of initial denaturation step at 95°C for 5 min followed by 35 cycles of denaturation at 95°C for 30 s, annealing at 60°C for 45 s, extension at 72°C for 1 min, and a final extention step at 72°C for 10 min.

Animal experiments

SK-Mel-147 human melanoma cells overexpressing miR-638 or scrambled-control were trypsinized and single cells were enriched by filtering them through a cell strainer. Melanoma cells (2 x 10^6) were then injected into the lateral tail vein of NSG (NOD *scid* IL2 receptor gamma chain knockout) mice [4]. On day 21 post injection, the mice were sacrificed and the lungs were dissected for macroscopic and microscopic histological analysis. After acquiring macroscopic images, the lungs were embedded into the tissue freezing medium and sectioned

for subsequent immunohistochemical analysis or stored at -80°C. Tissue sections (8 μm thickness) were stained with hematoxylin and eosin (H&E). The images were acquired using BZ-9000E microscopic system (Keyence) at 4-fold magnification, and the extent of metastasis was analysed with BZ-II analysis software (Keyence). All animal experiments were performed according to the institutional and state guidelines, and the committee of animal welfare of Saxony approved animal protocols used in this study (TVV 53/11).

Kinetic modelling of epigenetic deregulation of miR-638 promoter

The kinetic model accounts for the evolution in time of the expression levels for: miRNA-638 (represented in the model equations with the variable *miR*), transcriptionally active mRNA (*mA*) and protein (*A*) levels for AP-2α, transcriptionally active p53 ($p53^*$), MDM2 (*M2*) and an additional variable that accounts for expression of targets whose expression is regulated by both p53 and AP-2α (*Tgt*). Additionally, the model includes two tunable parameters accounting for the level of cellular stress (CS) and the methylation level of the AP-2α binding site in the promoter region of miR-638 (*MT*). The model is composed of six ordinary differential equations with the following structure:

$$\frac{dmiR}{dt} = k_{dm} \cdot \left(\frac{F_m}{(1+k_1 \cdot A)^2} - miR \right) \quad [1]$$

$$\frac{dmA}{dt} = k_{dmA} \cdot [F_{mA} - (1 + k_2 \cdot miR) \cdot mA] \quad [2]$$

$$\frac{dA}{dt} = k_{dA} \cdot (mA - A) \quad [3]$$

$$\frac{dp53^*}{dt} = k_{dp53} \cdot [CS - (1 + k_{m2}) \cdot M2 \cdot p53^*] \quad [4]$$

$$\frac{dM2}{dt} = k_{dm2} \cdot (p53^* - M2) \quad [5]$$

$$\frac{dTgt}{dt} = k_{dtg} \cdot (A \cdot p53^* - Tgt) \quad [6]$$

In case of miRNA-638**,** the model contains kinetic rates accounting for its AP-2α repressed synthesis (characterized by the parameters $F_{m,}$ *g* and k_1) and for its basal degradation (k_{dm}). For transcriptionally active AP-2α mRNA, we included kinetic rates accounting for its basal synthesis (F_{mA}), as well as for its basal degradation (k_{dmA}) and miR-638-mediated repression (k_2). For AP-2α, the model includes kinetic terms for its basal synthesis and degradation (k_{dA}). For p53, there were included terms describing the cellular stress mediated expression (*CS*), as well as its basal (k_{dp53}) and MDM2-enhanced degradation (k_{m2}). For MDM2, the model includes kinetic terms for its p53-mediated expression and its degradation (k_{dm2}). For the variable *Tgt*, we included a kinetic term accounting for the p53 and AP-2α mediated expression and another accounting for its degradation (k_{dtg}). The parameter k_1 depends on the methylation level of the AP-2α binding site (*MT*) according to the following function [5]:

$$k_1 = k_{1p} \cdot \left[\frac{100 - MT}{100}\right]^2$$

Parameters	Description of process	Value	Comments
k_{dm}	basal degradation of miR-638	0.029 hr^{-1}	Ref [6]
F_m	basal synthesis of miR-638	1 a.u.	normalization
k_{1p}	AP-2α mediated repression of miR-638	9 a.u.	assumed
MT	methylation level	[0,100] a.u.	tunable parameter
k_{dmA}	basal degradation of AP-2α mRNA	0.063 hr^{-1}	Ref [7]
F_{mA}	basal synthesis of AP-2α mRNA	1 a.u.	normalization
k_2	miR-638 mediated repression of AP-2α	9 a.u.	assumed
k_{dA}	basal degradation of AP-2α	0.023 hr^{-1}	Ref [8]
k_{dp53}	basal degradation of p53	0.0333 hr^{-1}	Ref [5]
CS	cellular stress level	[0,1] a.u.	tunable parameter
k_{m2}	MDM2-mediated repression of p53	9 a.u.	assumed
k_{dm2}	basal degradation of MDM2	0.0527 hr^{-1}	Ref [5]
k_{dtg}	basal degradation of p53/AP-2α target (i.e., p21)	0.1155 hr^{-1}	Ref [9]

Details for the kinetic model parameters of the mathematical modelling of the miR-638-mediated gene regulation of *TFAP2A*. The model variables were normalized between 0 and 1. The parameter value for k_{1p} was assigned such that for the maximum amount of AP-2α ($A=1$) and the minimum methylation level ($MT=0$) the expression level of miR-638 reduces to 1% of its maximum ($miR=0.01$). Similar assumptions were used for the parameters k_2 and k_{m2}.

Further details about the model parameters are given in the table above. The model was used to simulate the steady-state values of the model variables for different levels of cellular stress and methylation (*CS* and *MT*). Towards this end, we derived the equations accounting for the fixed points of the system, and computed them for given values of the tunable parameters *CS* and *MT* and chose the real stable solutions.

Supplementary References

1. Dvinge H, Bertone P.Dvinge H, Bertone P.HTqPCR: high-throughput analysis and visualization of quantitative real-time PCR data in R. Bioinformatics. 2009; 25(24):3325-3326.

2. Du P, Kibbe W A, and Lin S M.Du P, Kibbe W A, and Lin S M.lumi: a pipeline for processing Illumina microarray. Bioinformatics. 2008; 24(13):1547-1548.

3. Bhattacharya A, Schmitz U, Wolkenhauer O, Schonherr M, Raatz Y, and Kunz M.Bhattacharya A, Schmitz U, Wolkenhauer O, Schonherr M, Raatz Y, and Kunz M.Regulation of cell cycle checkpoint kinase WEE1 by miR-195 in malignant melanoma. Oncogene. 2013; 32(26):3175-3183.

4. Quintana E, Shackleton M, Sabel M S, Fullen D R, Johnson T M, and Morrison S J.Quintana E, Shackleton M, Sabel M S, Fullen D R, Johnson T M, and Morrison S J.Efficient tumour formation by single human melanoma cells. Nature. 2008; 456(7222):593-598.

5. Vera J, Schultz J, Ibrahim S, Raatz Y, Wolkenhauer O, and Kunz M.Vera J, Schultz J, Ibrahim S, Raatz Y, Wolkenhauer O, and Kunz M.Dynamical effects of epigenetic silencing of 14-3-3sigma expression. Mol.Biosyst. 2010; 6(1):264-273.

6. Kai Z S, Pasquinelli A E.Kai Z S, Pasquinelli A E.MicroRNA assassins: factors that regulate the disappearance of miRNAs. Nat.Struct.Mol.Biol. 2010; 17(1):5-10.

7. Schwanhausser B, Busse D, Li N, Dittmar G, Schuchhardt J, Wolf J, Chen W, and Selbach M.Schwanhausser B, Busse D, Li N, Dittmar G, Schuchhardt J, Wolf J, Chen W, and Selbach M.Global quantification of mammalian gene expression control. Nature. 2011; 473(7347):337-342.

8. Li M, Wang Y, Hung M C, and Kannan P.Li M, Wang Y, Hung M C, and Kannan P.Inefficient proteasomal-degradation pathway stabilizes AP-2alpha and activates HER-2/neu gene in breast cancer. Int.J.Cancer. 2006; 118(4):802-811.

9. Lai X, Schmitz U, Gupta S K, Bhattacharya A, Kunz M, Wolkenhauer O, and Vera J.Lai X, Schmitz U, Gupta S K, Bhattacharya A, Kunz M, Wolkenhauer O, and Vera J.Computational analysis of target hub gene repression regulated by multiple and cooperative miRNAs. Nucleic Acids Res. 2012; 40(18):8818-8834.
